# Editorial: Brain imaging techniques to measure treatment related effect

**DOI:** 10.3389/fnimg.2023.1238295

**Published:** 2023-07-26

**Authors:** Natalie M. Zahr, Assaf Harel, Efrat Sasson

**Affiliations:** ^1^Department of Psychiatry and Behavioral Sciences, School of Medicine, Stanford University, Stanford, CA, United States; ^2^Department of Psychology, Wright State University, Dayton, OH, United States; ^3^The Sagol Center for Hyperbaric Medicine and Research, Assaf Harofeh Medical Center, Ramle, Israel; ^4^WiseImage, Hod Hasharon, Israel

**Keywords:** brain imaging, neuroimaging, disease pathology, treatment efficacy, brain therapies

The collection of articles on the topic “*Brain imaging techniques to measure treatment related effects*” considers how therapy affects neuroimaging metrics. Although treatment evaluation is challenging because it presumes specific biomarkers of disease and potential changes in such markers as a function of successful treatment, non-invasive, longitudinal neuroimaging of quantitative changes is a viable option for tracking treatment efficacy and may provide insight into mechanisms of disease pathology.

Articles included in this Research Topic exemplify the breadth of this field and the wide range of neuroimaging tools available for treatment assessment. The Research Topic includes a variety of pathologies: renal disease, facial and back pain, epilepsy, and erectile disfunction; and a number of brain imaging tools such as structural magnetic resonance imaging (MRI), functional MRI (fMRI), diffusion weighted MRI (dwMRI), functional near-infrared spectroscopy (fNIRS), and positron emission tomography (PET).

Peng et al. evaluated the effects of a single hemodialysis treatment on brain morphology in patients with end-stage renal disease. Increases in gray and white matter volumes and concurrent decreases in CSF volumes suggest successful treatment. Rapid volume changes in response to a single treatment suggest mechanisms such as improved blood osmolarity (Peng et al.).

Zhang et al. used fNIRS to determine the effects of music as a treatment of myofascial pain syndrome. Appropriate music interventions seem to alleviate pain to some extent, possibly by reducing pain-associated brain activation in prefrontal and motor cortical regions (Zhang et al.).

Ailes et al. describe a case report using spinal cord stimulation to reduce pain in a patient suffering from failed back surgery syndrome. Reductions in reported pain were associated with decreased diffusivity in the insula as determined using dwMRI. This study suggests that spinal cord stimulation may reduce pain, but replication in a larger sample is necessary (Ailes et al.).

Mahmud et al. test the utility of the second generation PET ligand [^11^C]PBR28 as a marker to longitudinally track neuroinflammation. An inter-hemispheric asymmetry index was measured at two time points to evaluate the test-retest reliability of this marker in the hippocampus of healthy controls and individuals with epilepsy. This initial report provides sufficient data to permit estimates of power in future studies aimed at evaluating treatment efficacy in reducing volumes of distribution of this marker of neuroinflammation (Mahmud et al.).

Yang et al. studied erectile dysfunction to report abnormal patterns of activity in the posterior cingulate cortex, dorsolateral prefrontal cortex, supplementary motor area, and middle occipital gyrus, brain regions implicated in sexual inhibition. Electroacupuncture associated with improved scores on the International Index of Erectile Function (IIEF-5) and other questionnaires was associated with changes in the pattern of fMRI-derived low frequency fluctuations (Yang et al.).

The works included in this Research Topic highlight contemporary research efforts on identifying neuroimaging metrics that may be used for longitudinal tracking of therapy efficacy. We hope this Research Topic stimulates similar studies to use neuroimaging in the assessment of pharmacological and other treatment strategies on improving clinical outcomes in brain disease.

## Author contributions

All authors listed have made a substantial, direct, and intellectual contribution to the work and approved it for publication.

